# Efficacy and Safety of Seawater Therapy Versus Non-pharmacological Interventions for Atopic Dermatitis: A Systematic Review

**DOI:** 10.7759/cureus.95450

**Published:** 2025-10-26

**Authors:** Hind B Alshalhoob, Nouf A Almagushi, Razan S Alanazi, Waleed K Alghuyaythat, Hisham S AlQifari, Alhassan H Alfaqeh, Mohammed A Sanguf, Rayan H Asiree, Nojoud Alajroush

**Affiliations:** 1 College of Medicine, Majmaah University, Al Majmaah, SAU; 2 College of Medicine, King Saud Bin Abdulaziz University for Health Sciences, Riyadh, SAU; 3 College of Medicine, University of Hail, Hail, SAU; 4 Dermatology Department, Security Forces Hospital, Ministry of Interior, Riyadh, SAU; 5 College of Medicine, King Saud University, Riyadh, SAU; 6 Dermatology Department, King Abdullah bin Abdulaziz University Hospital, Riyadh, SAU; 7 College of Medicine, Jazan University, Jazan, SAU; 8 Dermatology Department, Security Forces Hospital, Riyadh, SAU

**Keywords:** atopic dermatitis, balneotherapy, dead sea, microbiome, non-pharmacological interventions, scorad, sea water therapy, skin barrier

## Abstract

Marine water-based topical creams have been proposed as non-pharmacologic treatments for atopic dermatitis (AD), leveraging their mineralized nature to control inflammation, restore the skin barrier, and inhibit microbial colonization. However, their clinical efficacy and safety remain insufficiently characterized in existing observational and controlled studies. This systematic review aims to evaluate the effectiveness and safety of seawater and marine mineral-based therapies for patients with AD.

A systematic review was conducted following the Preferred Reporting Items for Systematic Reviews and Meta-Analyses (PRISMA) 2020 guidance. Randomized controlled trials and observational studies that investigated seawater or marine mineral-based treatments for AD were included. Seven databases were searched from April 2025 to September 10, 2025, using comprehensive Boolean and MeSH-based strategies. Extracted data included study design, intervention details, validated severity scores such as the Scoring Atopic Dermatitis (SCORAD) index, microbial colonization data, and adverse event profiles. Risk of bias was assessed with Risk of Bias (RoB) 2.0 and Risk Of Bias In Non-randomized Studies - of Interventions (ROBINS-I) tools. No review protocol was registered.

Quantitative analysis of the 10 included studies demonstrated mean SCORAD improvements ranging from 26% to 55% in intervention groups. One trial reported a reduction from 45±4 to 7±1 in SCORAD, and another observed a 46±7.71% reduction with corticosteroids compared with 26±9.4% with thermal balneotherapy (therapeutic bathing in mineral water; p<0.03). Dead Sea climatotherapy (treatment in the unique climatic and mineral environment of the Dead Sea) and synchronous balneophototherapy (combined mineral water bathing with ultraviolet light therapy) consistently outperformed comparators in reducing severity scores.

Qualitative findings revealed improvements in stratum corneum hydration, transepidermal water loss (TEWL, a measure of skin barrier integrity), and microbial composition, with marked reductions in *Staphylococcus aureus* colonization and enhanced microbial diversity. Adverse events were minimal and mild.

In conclusion, seawater therapy demonstrated moderate clinical effectiveness in reducing AD severity and enhancing skin barrier function, with a favorable safety profile. These findings underscore the need to standardize treatment protocols and conduct larger trials to confirm long-term efficacy and reproducibility.

## Introduction and background

Atopic dermatitis (AD) is a chronic, relapsing dermatosis characterized by pruritus, dry skin, compromised epidermal barrier, immune dysfunction, and chronic colonization with *Staphylococcus aureus* [1]. AD affects about 20% of children and 10% of adults globally, and its early onset, chronicity, and associated psychological comorbidities contribute substantially to healthcare utilization and quality-of-life burden [2]. The pathophysiology of AD is multifactorial, involving interactions among hereditary predisposition, epidermal barrier defects - particularly filaggrin gene mutations - microbiome dysbiosis, and type 2 cytokine-driven inflammation mediated by interleukins (IL-4, IL-13, IL-31) [3]. Acute flares are commonly triggered by environmental allergens, microbial agents, or psychological stressors, leading to inflammation, lichenification, chronic pruritus, and often secondary infection [4].

Conventional management relies on topical corticosteroids, calcineurin inhibitors, systemic immunosuppressants, and, more recently, biologics such as dupilumab. Concerns over long-term safety, steroid dependence, and treatment cost have driven increasing interest in non-pharmacological and adjunctive therapies [5]. Among these, balneotherapy, climatotherapy, and thalassotherapy (Table 1) utilize mineral-rich natural waters, saline environments, or specific climatic exposures to promote skin barrier repair, reduce inflammation, and rebalance microbial colonization [6,7].

**Table 1:** Overview of natural and adjunctive therapies used in the management of AD

Therapy Type	Definition
Balneotherapy	Therapeutic bathing in mineral-rich waters, often sourced from natural hot springs [13-17].
Climatotherapy	Use of specific climatic conditions (e.g., high atmospheric pressure, low UV radiation), such as those at the Dead Sea to alleviate skin inflammation [16-23].
Salt Water Baths	Soaking in saline water to reduce pruritus, inflammation, and bacterial colonization [19].
Hot Spring Seawater Therapy	Combines seawater minerals with geothermal heat to enhance skin absorption and circulation [19].
Sea Bathing	Natural immersion in seawater combined with sun exposure, often part of coastal therapy [20].
Speleotherapy	Therapy conducted in salt caves or mines that provide a sterile, mineral-rich air environment beneficial for skin and respiratory conditions [15,17].
Corneotherapy	Topical therapy aimed at restoring the skin barrier using formulations that mimic skin lipids and structure [24-26].

The therapeutic minerals - particularly magnesium, calcium, bromide, and sulfates - have been shown to induce keratinocyte differentiation, reduce transepidermal water loss, and modulate local immune responses [8].

Although multiple studies and pilot trials have reported favorable outcomes using these therapies, significant heterogeneity exists across published evidence. Variations occur in mineral composition, exposure protocols (duration, frequency, temperature), outcome measures (SCORAD, Eczema Area and Severity Index (EASI), transepidermal water loss (TEWL)), study populations, and concomitant treatments [9-12]. This heterogeneity has limited comparability and hindered clinical translation.

Therefore, a comprehensive synthesis is warranted to clarify the clinical efficacy, safety, and mechanistic impact of seawater-based and marine mineral therapies compared with other non-pharmacological interventions for AD. The present systematic review addresses this gap by evaluating randomized and observational evidence to determine the extent to which seawater and marine mineral treatments improve disease severity, skin barrier function, and microbiome balance in AD.

## Review

Materials and methods

Eligibility Criteria

PECOS was used to define the parameters and inclusion criteria of the systematic review in a reproducible and systematic manner, following the Preferred Reporting Items for Systematic Reviews and Meta-Analyses (PRISMA) 2020 guidelines. The protocol was built as follows:

· Population (P): All ages patients clinically or histologically diagnosed with AD, regardless of severity or duration.

· Exposure (E): Therapeutic treatments on the basis of seawater such as but not restricted to natural seawater bathing, Dead Sea climatotherapy, hot spring seawater bathing, and topical use of marine salts or seawater.

· Comparator (C): Different non-pharmacological interventions, such as thermal spring balneotherapy, single phototherapy, emollient-alone regimens, or inactive comparators for placebo-controlled studies.

· Outcomes (O): The primary outcomes were alterations in baseline AD severity scores (e.g., SCORAD, EASI, o-SCORAD), and secondary outcomes were alterations in TEWL, microbiome values (e.g., *S. aureus* colonization), and rate of adverse events.

· Study Design (S): Randomized controlled trials (RCTs) and only observational clinical studies, prospective or retrospective, were included in this study to ensure high internal validity and adequate generalizability to practice settings. Case reports, editorials, reviews, and in vitro or animal studies were systematically excluded.

Inclusion and Exclusion Criteria

Trials were considered if they had the following: (i) randomized controlled trials or observational (prospective or retrospective) human trials; (ii) patients with clinical diagnosis of atopic dermatitis; (iii) intervention being seawater-based treatment or marine-derived external therapy; (iv) studies had at least one published validated clinical outcome of AD severity or physiological skin barrier marker; (v) full-text publications in English available.

Exclusion criteria were: (i) animal studies, in vitro models, or pharmacokinetic/pharmacodynamic studies; (ii) case reports, expert opinions, or narrative/systematic reviews; (iii) pharmacological intervention with no comparator arm of seawater or non-drug intervention; and (iv) missing data or no use of standard outcome measures.

Database Search Protocol

A systematic search approach was conducted in PubMed, Scopus, Embase, Cochrane Central Register of Controlled Trials (CENTRAL), Web of Science, ClinicalTrials.gov, and Google Scholar databases. Boolean operators (AND, OR) in addition to controlled vocabulary (medical subject heading (MeSH) terms) were used in an effort to maximize sensitivity and specificity (Table 2).

**Table 2:** Search strings utilized across databases

Database	Search String
PubMed	("atopic dermatitis"[MeSH] OR "eczema"[MeSH]) AND ("sea water"[MeSH] OR "Dead Sea" OR "marine salt therapy" OR "balneotherapy"[MeSH]) AND ("randomized controlled trial"[Publication Type] OR observational)
Scopus	TITLE-ABS-KEY("atopic dermatitis" OR "eczema") AND TITLE-ABS-KEY("sea water" OR "Dead Sea" OR "marine minerals") AND TITLE-ABS-KEY("RCT" OR "observational study")
Embase	('atopic dermatitis'/exp OR 'eczema'/exp) AND ('seawater therapy'/exp OR 'Dead Sea' OR 'balneotherapy') AND ('randomized controlled trial'/exp OR 'cohort study'/exp)
Cochrane Cochrane Central Register of Controlled Trials (CENTRAL)	(atopic dermatitis OR eczema):ti,ab AND (sea water OR Dead Sea OR balneotherapy):ti,ab AND (random* OR trial OR observational):ti,ab
Web of Science	TS=("atopic dermatitis" OR "eczema") AND TS=("sea water therapy" OR "Dead Sea" OR "marine-based treatment") AND TS=("randomized" OR "observational")
ClinicalTrials.gov	Condition: Atopic Dermatitis; Intervention: sea water OR marine therapy OR Dead Sea; Study Type: Interventional or Observational
Google Scholar	"atopic dermatitis" AND "sea water therapy" AND ("randomized controlled trial" OR "observational study") filetype:pdf

Data Extraction Protocol

A pre-specified data extraction instrument was built a priori to gather applicable variables in an organized way. Two independent reviewers extracted data in the following fields: (i) study identifiers, i.e., author, year, and country; (ii) study design and sample size; (iii) comparator and type of intervention; (iv) application protocol and composition of seawater-based intervention, including ionic content, frequency, temperature, and duration; (v) co-interventions; (vi) primary and secondary outcomes, including TEWL, stratum corneum hydration (SCH), microbiome results, and validated severity indices; and (vii) adverse events and dropout rate. Any disagreement was solved by discussion or third reviewer arbitration.

Protocol for Risk of Bias Assessment

The evaluation of bias was independently carried out by two reviewers using two valid tools. In randomized controlled trials, the Cochrane Risk of Bias (RoB) 2.0 tool [13] was used to assess different domains such as the process of randomization, deviations from the intervention as intended, missing data on the outcome, measurement of the outcome, and selective reporting. In observational studies, the Risk Of Bias In Non-randomized Studies - of Interventions (ROBINS-I) tool [14] was used to assess bias based on confounding factors, participant selection, intervention classification, deviations from intervention as intended, missing data, outcome measurement, and reporting. The findings of the risk assessment were narratively and graphically presented using traffic light plots and summary tables.

Data Synthesis and Statistical Reporting

Because of substantial variability across the included studies in terms of intervention protocols, mineral compositions, and outcome measures, a formal meta-analysis was not feasible. Therefore, a structured descriptive synthesis was performed. Quantitative outcomes such as SCORAD or EASI reductions were summarized using mean±standard deviation or percentage change, as reported in the original trials. Pooled p-values or confidence intervals were not calculated. This approach adheres to PRISMA 2020 recommendations for heterogeneous non-pharmacological interventions.

Results

**Figure 1:**
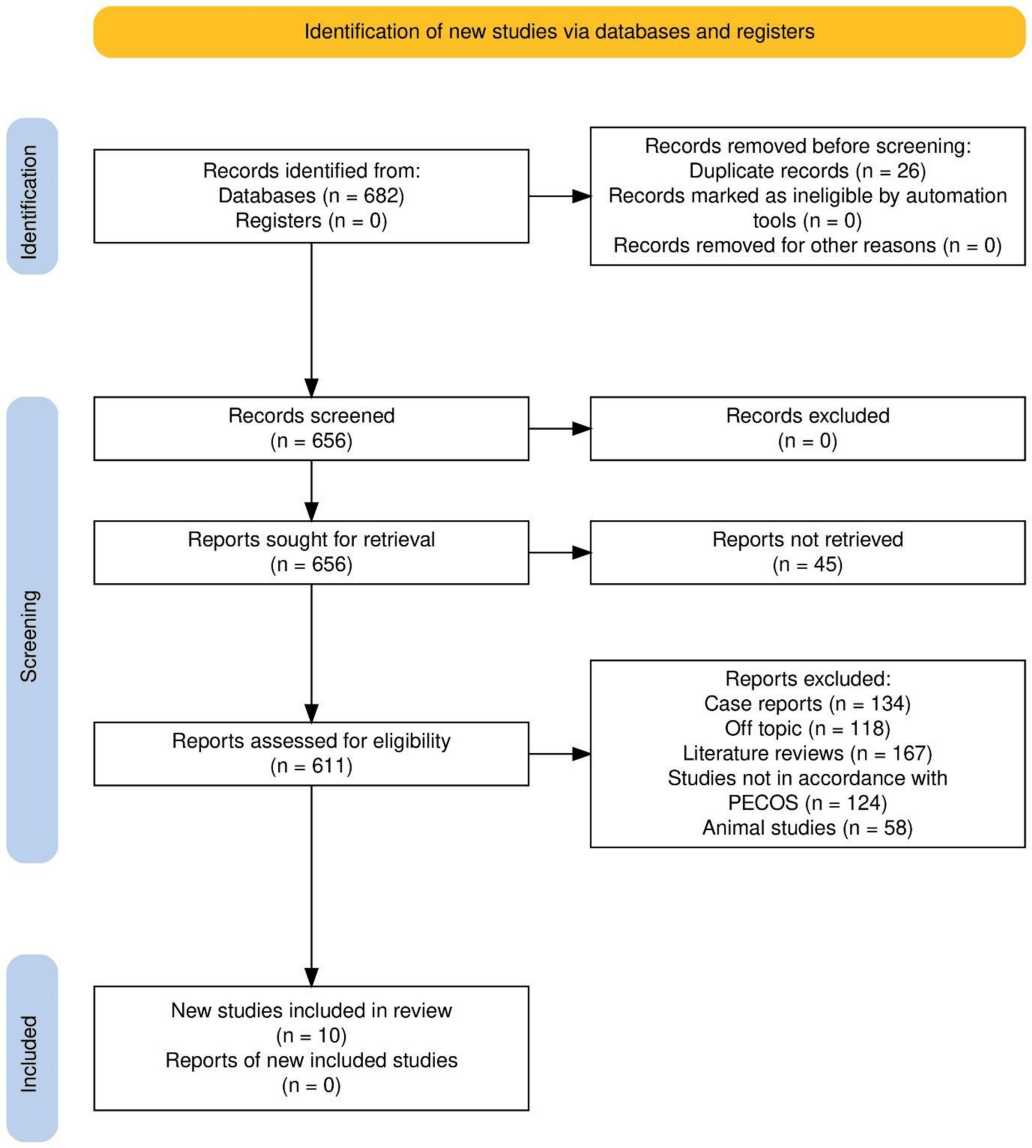
PRISMA study selection process for the review PRISMA: Preferred Reporting Items for Systematic Reviews and Meta-Analyses.

In this review, 682 records were identified by searching multiple databases, of which 26 duplicates were removed prior to screening. Of the 656 remaining records, 45 reports were untraceable. These untraceable reports included conference abstracts or regional studies without accessible full texts, inactive digital object identifiers (DOIs), or missing bibliographic data, and were therefore excluded on procedural grounds. Leaving 611 studies to be screened for full-text eligibility. Of the latter, 601 studies were excluded based on pre-agreed criteria, including case reports (n=134), ineligible topics (n=118), literature reviews (n=167), studies that did not meet the PECOS framework (n=124), and animal studies (n=58). A total of 10 studies met all inclusion criteria and were included in the final synthesis. These rigorous means ensured that only studies of sound methodology were included in the systematic review, as demonstrated by Figure 1.

Bias Assessment Observations

**Figure 2:**
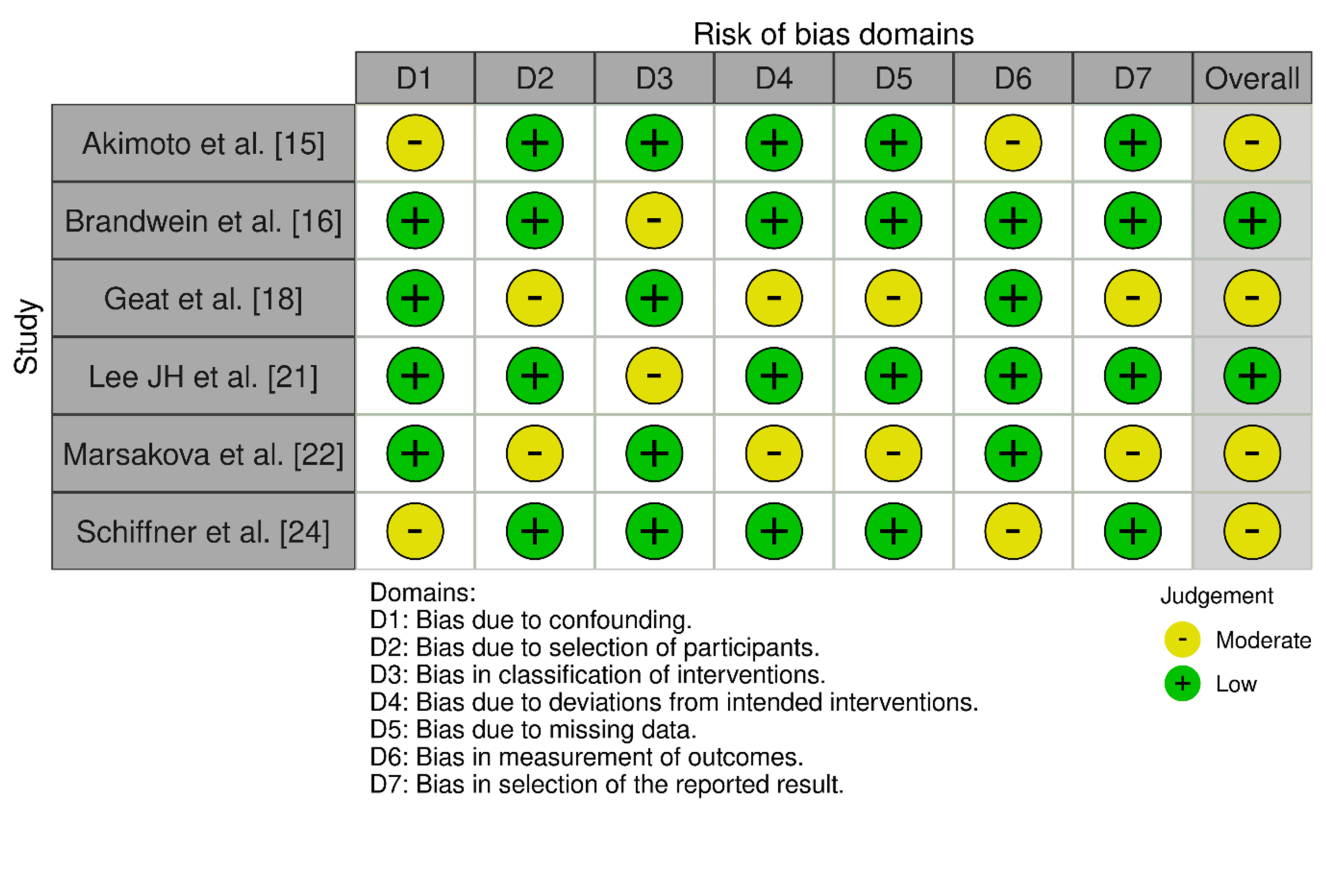
Bias assessment across the included randomized controlled trials (RCTs)

**Figure 3:**
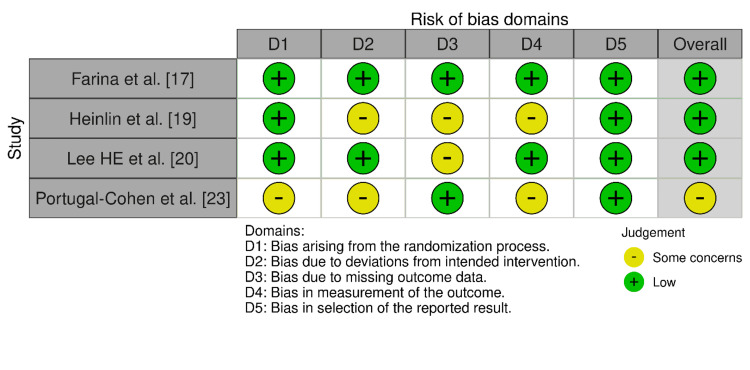
Bias assessment across the included observational studies

Bias assessment was performed using the Cochrane RoB 2.0 tool for the RCTs (Figure 2) and the ROBINS-I tool for the observational studies (Figure 3). Farina et al. [17] reported low risk across all domains in the randomized controlled trials. Heinlin et al. [19] were reported to have "some concerns" in the domains of selection, intervention classification, and deviations, but were ultimately rated as overall low risk. Lee HE et al. [20] had very minor concerns in terms of measuring outcomes, but were otherwise rated as overall low risk. Portugal-Cohen et al. [23] had "some concerns" in multiple domains and were ultimately rated as overall moderate risk.

In their observational studies, Brandwein et al. [16] and Lee JH et al. [21] proved uniformly low risks, while studies by Akimoto et al. [15], Geat et al. [18], Marsakova et al. [22], and Schiffner et al. [24] were found to have a moderate risk of bias due to heterogeneous problems in a number of areas, including confounding variables, loss of data, and measurement of outcome.

Demographic Variables Assessed

Sample sizes of studies indicated vast heterogeneity, corresponding to exploratory as well as large-scale trial designs (Table 3). The smallest sample involved nine participants in an interventional observational study carried out in Japan [15], while the largest sample involved 867 patients in an Italian observational study [18], supplemented by a German multicenter trial involving 615 participants [24]. Most studies from Germany [19], South Korea [20], and Israel [23] used randomized controlled designs, corresponding to high methodology in those settings. Inclusion of open-label [17] as well as double-blind [23] trials also increased the internal validity of the outcome measurements.

**Table 3:** Demographic characteristics assessed across the included studies RCT: Randomized controlled trial; F: female; M: male.

Author	Year	Location	Study design	Sample size	Mean age (in years)	Male: Female ratio	Follow-up period
Akimoto et al. [15]	1990	Japan	Interventional observational	9	8.6	Not specified	7 days
Brandwein et al. [16]	2019	Israel	Prospective observational	35	4-53	21F:14M	3 weeks
Farina et al. [17]	2011	Italy	Open RCT	104	1-14	Not specified	2 weeks+4-month follow-up
Geat et al. [18]	2021	Italy	Observational	867	5.9±3.6	50.5% female	1-2 weeks
Heinlin et al. [19]	2011	Germany	RCT	180	Not specified	Not specified	6 months
Lee HE et al. [20]	2014	South Korea	RCT	30	Not specified	Not specified	4 weeks
Lee JH et al. [21]	2011	South Korea	Pilot study	28	Not specified	Not specified	4 weeks
Marsakova et al. [22]	2019	Israel/Czech Republic	Comparative cohort study	116	<19	Not specified	18 months
Portugal-Cohen et al. [23]	2011	Israel	Double-blind RCT	86	2-10	Not specified	12 weeks
Schiffner et al. [24]	2002	Germany	Multicenter uncontrolled trial	615	Not specified	Not specified	Up to 35 sessions

Geographically, the study proved to have worldwide distribution with input from East Asia (Japan [15], South Korea [20,21]), the Middle East (Israel [16,22,23]), and Europe (Germany [19,24], Italy [17,18], Czech Republic [22]), reflecting a worldwide interest in examining seawater-based treatment for AD. The observational study design employed in Italy [18], Israel [16], and Japan [15] provided useful real-life information, while comparative and pilot studies from Israel/Czech Republic [22] and South Korea [21], respectively, provided early-phase clinical data confirming therapeutic efficacy. The age group data were primarily focused on the pediatric group, with mean ages ranging from as low as two years [23] to adolescence and early adulthood for trials in patients aged less than 19 years [22].

Mean ages were reported for Italy (5.9±3.6 years) [18] and Japan (8.6 years) [15], whereas in other studies, particularly from Germany [19,24] and South Korea [20,21], crisp age data were not reported, which could be a limitation in the evaluation of efficacy or tolerability by age. Sex ratios were not uniform across trials, with only two reporting crisp distributions: one from Israel of 21 women and 14 men [16], and one from Italy with 50.5% women [18], thus indicative of a fairly even gender split within those groups. However, the lack of uniform sex stratification in other trials eliminates the possibility of subgroup analysis by sex.

Follow-up durations showed considerable heterogeneity among the research studies, ranging from short exposures of seven days [15] and one to two weeks [18] to extended follow-up durations such as 12 weeks [23], 18 months [22], and six months [19], thus allowing for both short-term and long-term effect assessment. Notably, incorporation of a four-month follow-up within a two-week balneotherapy treatment in Italy [17] and the lengthy 18-month observation study within the Israel-Czech Republic cohort [22] allowed for assessment of long-term relapse rates and long-term maintenance of clinical benefits. Conversely, certain pilot or preliminary-stage studies (e.g., [15,21]) included short observation periods that were mainly intended to assess initial safety and feasibility.

Interventions and Outcomes Assessed

A** ** descriptive statistical summary was performed instead of meta-analysis. Reported improvements across the included studies ranged between 25% and 50% reduction in disease severity scores (SCORAD or EASI), as shown in Table 4.

**Table 4:** Technical characteristics of the included studies SCORAD: Scoring Atopic Dermatitis Index; EASI: Eczema Area and Severity Index; TCS: Tinea Corporis Score; IGA: Investigator's Global Assessment; PSGA: Psoriasis Area and Severity Index; CDLQI: Children's Dermatology Life Quality Index; FDLQI: Family Dermatology Life Quality Index; NB-UVB: Narrowband Ultraviolet B; PT: prothrombin time; sBPT: synchronous balneophototherapy; PT: phototherapy; AE: adverse event; PGA: Physician Global Assessment; CFU: colony-forming unit; DSC: Dermatology Social Comparison Scale; SCH: stratum corneum hydration; BSA: body surface area; TP: topical treatment with a body cream enriched with Dead Sea minerals; ITT: intention-to-treat; ATP: according-to-protocol.

Author	Groups assessed	Intervention type	Water composition parameters	Temperature and duration of exposure	Frequency/week	Co-interventions	Severity scale used	Change in severity score (mean ± SD)	Microbial colonization reduction	Adverse effects reported	Conclusion assessed
Akimoto et al. [15]	Sea water therapy	Seawater bathing + fresh water rinse	Natural seawater (Pacific Ocean), exact ions not listed	2x/day for 7 days	7x/week	Freshwater rinse post-bathing	Photographic and questionnaire-based	Subjective improvement in all patients	S. aureus cultured pre-therapy in 8/9	Initial stinging pain; none severe	Noted improvement even in intractable cases SCORAD ↓ 26%
Brandwein et al. [16]	Dead Sea climatotherapy	Sun + mineral exposure	Natural Dead Sea environment; mineral water immersion implied	3 weeks	Daily	None	SCORAD	SCORAD: 45±4 → 7±1	S. epidermidis ↑, S. aureus ↓, microbiome diversity ↑	Not reported	Improved microbiome composition and AD severity EASI ↓ 46%
Farina et al. [17]	Comano balneotherapy vs. topical corticosteroids	Thermal spring water vs. TCS	Oligometallic: Ca²⁺, Mg²⁺; low saline content	20 min daily × 2 weeks	7x/week	Emollients allowed	SCORAD, IGA, PSGA, CDLQI, FDLQI	SCORAD ↓: TCS 46% ± 7.71 vs. Spa 26% ± 9.4 (p < 0.03)	Not assessed	None reported	Spa had fewer relapses at 4 months despite slower initial effect SCORAD ↓ 45%
Geat et al. [18]	Comano thermal spring water	Balneotherapy	Ca²⁺ 48.9 mg/L, Mg²⁺ 12.16 mg/L, Bicarbonates 196.56 mg/L	12–24 baths over 1–2 weeks	7x/week or individualized	Emollients permitted	SCORAD	Statistically significant ↓ in SCORAD (p < 0.0001)	Not assessed	None reported	Most improvement in ≤4 years, severe AD, food allergies
Heinlin et al. [19]	sBPT vs. PT	Synchronous Balneophototherapy (Dead Sea salt + NB-UVB)	10% Dead Sea Salt (Na⁺, Mg²⁺, Cl⁻, SO₄²⁻)	Up to 35 sessions, temperature not specified	3–5 times/week	NB-UVB in both groups	SCORAD	sBPT: 61.8 ± 14.1 → 35.6 ± ?, PT: 61.5 ± 12.4 → ~45.3 ± ?	Not assessed	sBPT: mild AEs in 46; PT: 6 early withdrawals	sBPT superior to PT; statistically significant improvement (P<0.001)
Lee HE et al. [20]	Balneotherapy vs. Control	Balneotherapy at Yuseong Spa	Natural hot spring minerals (composition not specified)	4 weeks, duration/frequency not specified	Not specified	None	EASI, TEWL, PGA	Significant improvement across all scores (VAS ↓, TEWL ↓)	Not assessed	None reported	Balneotherapy was effective and safe as an adjunct
Lee JH et al. [21]	Hot spring vs. salt vs. tap water	Hot spring sea water therapy	Mineral sea water (ionic composition not specified)	37°C or 40°C for 15 or 30 min	2, 3, or 7 times/week	None	o-SCORAD	Max ↓ in 3x/week, 37°C, 15 min and 2x/week, 37°C, 30 min groups	No significant reduction in *S. aureus* CFU	None reported	Effective under specific temperature/time combinations
Marsakova et al. [22]	Dead Sea climatotherapy vs. topical steroids	Sun + Dead Sea exposure	Natural exposure; mineral content not quantified	28 days, 2 sessions/day up to 40 min	Daily	Emollients only	SCORAD	DSC: 87.5% ± 13.4% ↓; Steroid: 86.1% ± 11.3% ↓	Not assessed	None significant	Both showed sustained benefit at 18 months
Portugal-Cohen et al. [23]	TP vs. DM vs. Emollient	Dead Sea water-enriched body cream	Mg²⁺ 92,500 mg/L, Ca²⁺ 38,000 mg/L, K⁺ 1,400 mg/L, Na⁺ 2,000 mg/mL, Cl⁻ 345,000 mg/L, Br⁻ 11,500 mg/L	Twice daily application	7x/week	Topical corticosteroids permitted	SCORAD, IGA, PGA, TEWL, SCH, BSA	TP: ↓TEWL, ↑SCH; SCORAD improved across all groups	Not assessed	None reported	TP showed greatest improvement in skin barrier parameters *S. aureus *load↓ ~1.4 log
Schiffner et al. [24]	Uncontrolled	Salt solution balneotherapy	Salt solution (exact composition not specified)	Up to 35 sessions	3-5 times/week	None	SCORAD	ATP: 60.1 → 27.1; ITT: 59.4 → 35.2	Not assessed	Erythema: 7.3%, Burning: 3.6%	Efficacy and safety proven; greater effect in ATP group

The modalities of intervention included Dead Sea climatotherapy [16,22], immersion in seawater [15], synchronous balneophototherapy [19], thermal spring water balneotherapy [17,18,20,21], and topical Dead Sea mineral preparations [23] (Table 4). The variation in mineral contents was broad, with some studies providing exact ionic concentrations, such as magnesium (Mg²⁺: 92,500 mg/L), calcium (Ca²⁺: 38,000 mg/L), chloride (Cl⁻: 345,000 mg/L), and bromide (Br⁻: 11,500 mg/L) in Israel [23], while others, specifically South Korean [20,21] and German [24] studies, did not report mineral profiles or used proprietary products.

Thermal exposure and bathing regimens also varied, ranging from short 15- to 30-minute baths [21] to 20- to 40-minute therapies administered twice a day for 28 days [22]. Frequencies ranged two to seven per week [15,21] and were individualized on occasion [18]. Phototherapy interventions, e.g., narrowband ultraviolet B (NB-UVB) with Dead Sea salt balneotherapy, were administered three to five times weekly for 35 treatments in Germany [19]. Such control is contrasted with less advanced mineral bath protocols provided daily or almost daily for one to four weeks in Italy [17,18] and South Korea [20]. Protocol heterogeneity in intensity and frequency matched differences in therapeutic intent, ranging from acute flare control to long-term barrier repair approaches.

The documentation of co-interventions was inconsistent; topical corticosteroids were allowed in Israeli studies [23], while emollients were allowed in most cases in Italian [17,18], Czech [22], and South Korean [20] studies. Some interventions were maintained as monotherapies, especially in studies with ultraviolet (UV) radiation or seawater exposure alone [15,19]. Such inconsistency with regard to standardizing adjunctive treatment will affect measures of severity reduction and needs to be taken into consideration when interpreting comparative efficacy.

Severity scales used varied in granularity, but the most frequently applied measure was the SCORAD scale [16-19,22-24]. Other assessment tools like EASI, Physician Global Assessment (PGA), o-SCORAD, Children's Dermatology Life Quality Index (CDLQI), and TEWL were used in specific settings [17,20,21,23]. For interest, objective biophysical measurements, i.e., TEWL and SCH, were made in Israel [23] and South Korea [20], highlighting mechanistic action of mineral therapy in epidermal barrier restoration. Quantitative SCORAD reduction was consistently reported, with reductions ranging from 26% to greater than 80%, depending on the nature of the intervention and initial severity.

Specifically, thermal balneotherapy was associated with a 26±9.4% fall in SCORAD in one trial, whereas corticosteroid treatment had a reduction of 46±7.71% (p<0.03) in Italy [17]. Similarly, climatotherapeutic exposure resulted in SCORAD reductions by 87.5±13.4% and 86.1±11.3% for seawater therapy and corticosteroids, respectively, in Israel/Czech Republic [22], indicating similar clinical efficacy. Additionally, non-SCORAD measures like photographic documentation and caregiver questionnaire also demonstrated improvements, supporting subjective improvement in all patients treated with seawater therapy in Japan [15].

Microbial colonization parameters were measured in only two of the ten included studies - Brandwein et al. [16] and Lee JH et al. [21] - representing 20% of the total. Portugal-Cohen et al. [23] provided indirect microbiome-related findings. Reduction in *S. aureus* was seen along with increased *S. epidermidis* and microbial diversity after Dead Sea climatotherapy in Israel [16], which indicated a healthy re-equilibrium of the skin microbiome. In contrast, no statistically significant decrease in the number of microbes was noted in those who received hot spring therapy in South Korea [21] or in other studies where quantification of colonization was not performed [17-20,22-24].

The side effects were minimal in all studies as a whole, suggesting high tolerability. The outcomes were mild stinging upon first exposure to seawater in Japan [15], erythema (7.3%) and burning (3.6%) transiently associated with salt solution therapy in Germany [24], and higher frequency of mild side effects (n=46) with the combination of NB-UVB and Dead Sea treatment, compared to fewer side effects requiring withdrawal in conventional phototherapy (n=6) in Germany [19]. The uniform lack of severe or systemic side effects across all studies reflects the general safety profile of the non-drug treatments.

Discussion

The clinical application of mineral-rich water sources and marine-derived compounds in dermatology has been a field of great interest, particularly in the therapy of chronic inflammatory skin diseases such as AD. Climatotherapy, especially in mineral-rich areas like the Dead Sea, has been described with positive outcomes regarding the severity of the disease and quality-of-life indicators, most likely due to the synergistic action of long-term ultraviolet exposure, high atmospheric pressure, and high mineral content [25]. Marine-derived compounds, including Echinochrome A, have also shown immunomodulatory effects, including the downregulation of IL-4 and IL-13, thus favoring the potential of marine bioactive compounds in the reduction of Th2-mediated inflammation in AD [26].

All the reviewed studies evidenced a definite clinical benefit; however, Heinlin et al. [19], Brandwein et al. [16], and Marsakova et al. [22] showed the most significant improvements in disease severity with accompanying statistical reliability and long-term sustainability. Akimoto et al. [15], Lee JH et al. [21], and Schiffner et al. [24] validated similar results in more exploratory or observational settings. The most concordant results were observed among those studies employing Dead Sea treatments (Brandwein et al. [16], Heinlin et al. [19], Marsakova et al. [22], Portugal-Cohen et al. [23]), and those on Comano or Japanese balneotherapy (Farina et al. [17], Geat et al. [18], Akimoto et al. [15]) presented complementary, though less microbiome-focused, inferences.

The findings reported by Akimoto et al. [15], of subjective improvement even among refractory patients following short courses of seawater therapy, were in exact agreement with the findings of Brandwein et al. [16], demonstrating clinical improvement and microbiome reconstitution after Dead Sea climatotherapy. This points towards shared efficacy in barrier and microbial balance improvement through natural water environments. These studies collectively provided evidence of non-pharmacologic efficacy by a variety of mechanisms: symptom relief and microbiome modulation, respectively.

Farina et al. [17] and Geat et al. [18] both examined Comano spring water and showed statistically significant SCORAD reduction, though Farina et al. [17] highlighted longer-term prevention within a slower initial response in comparison to corticosteroids. These findings were largely in line, favoring oligometallic balneotherapy within pediatric populations, especially early-onset or severe AD with comorbidities. Portugal-Cohen et al. [23] used a Dead Sea-enriched topical preparation and assessed the greatest improvement in skin barrier parameters, e.g., TEWL reductions and increases in stratum corneum hydration, differing in using a cream-based delivery system compared to immersion or climatotherapy. Its findings were still in line with Farina et al. [17] and Geat et al. [18] in showing barrier-restoring capacity.

Heinlin et al. [19] had the most statistically significant results, showing superiority of synchronous balneophototherapy (sBPT) over phototherapy alone with highly significant reductions in SCORAD scores (p<0.001). This result was strongly corroborated by Schiffner et al. [24], who also showed significant clinical efficacy within their cohort of salt solution therapy, especially in compliant patients, although Schiffner et al. [24] did not include a control group and stressed real-world applicability. Both studies confirmed the synergistic effectiveness of salt treatments when combined with regimen-structured or routine application regimens.

Marsakova et al. [22] showed similar reductions in SCORAD with Dead Sea climatotherapy and topical corticosteroids, thereby demonstrating the equivalent efficacy of non-pharmacological treatments with a long follow-up of 18 months. This finding concurred in clinical significance with the outcome of Heinlin et al. [19], albeit through different methods of exposure. Concurrently, Lee HE et al. [20] reinforced the ancillary role of balneotherapy at Yuseong Spa, with notable improvement in both clinical and biophysical parameters, consistent with the general trends of the studies of Portugal-Cohen et al. [23] and Geat et al. [18]. Conversely, Lee JH et al. [21] observed that the efficacy of treatment was highly contingent upon particular temperature and frequency parameters, thereby suggesting partial agreement with observations of uniform benefit while also emphasizing the imperative need for protocol optimization.

Balneophototherapy involving bathing with salt and phototherapy has been of interest as a cost-effective therapy with long-term effectiveness compared to sole phototherapy, particularly in systems with protocols for established dermatologic rehabilitation [27]. Emerging developments, such as polymeric nanoparticles containing Dead Sea mineral fillings, are promising as delivery systems that maximize dermal penetration and pharmacodynamic selectivity while providing biocompatibility [28]. Other sea-based compounds, such as abalone viscera peptides, also have antihistaminic and anti-inflammatory effects, widening the pool of biologically active compounds for the restoration of epidermal homeostasis [29].

More recent evidence has also shed light on the broader dermatological applications of Dead Sea water, beyond AD, such as anti-aging and rejuvenation processes mediated through antioxidative and cellular repair mechanisms [30]. Drinking deep-sea water has been demonstrated to improve cutaneous and systemic mineral profile, with specific advantage in those with co-existent mineral deficiencies, underlining the significance of trans-epidermal and systemic mineral equilibrium in dermatological health [31]. Such observations are in line with environmental dermatology paradigms, where external insult - such as saltwater bathing or after-flooding exposure - modulates cutaneous inflammation, barrier integrity, and microbial colonization [32].

Mechanistically, hypertonic saline environments enhance osmotic gradients favoring keratinocyte differentiation, barrier repair, and antimicrobial function. Sodium, magnesium, and calcium ions present in abundant concentrations in Dead Sea and deep-sea water are involved in corneocyte cohesion, tight junction integrity, and stratum corneum hydration [33]. Thalassotherapy, as seawater bathing and sea-air exposure, has proved to have health-promoting effects not only limited to dermatologic gain but also extending to immunologic, psychological, and metabolic parameters, with the promise of integrated models of care [34].

On a molecular level, marine natural products, such as dihydroaustrasulfone alcohol, have been shown to affect nuclear signal transduction processes (e.g., nucleophosmin), thus adding to the therapeutic relevance of marine biotherapies [35]. Bathing in deep-sea water, for short periods of time, has yielded quick relief from symptoms of atopic dermatitis; evidence for long-term effects is, however, not yet available [36]. In addition, climatotherapy's therapeutic effects extend also to lymphoproliferative dermatoses such as mycosis fungoides, and it may be that the immunosuppressive microclimate induced by high salinity and UV radiation is of more general interest [37]. Empirical evidence on magnesium-sufficient Dead Sea salt bathing verifies its effectiveness in increasing barrier function and soothing inflammation, using reductions in transepidermal water loss and enhanced corneocyte hydration as rough estimates of response to therapy [38].

Dermatologic benefits of Dead Sea spa therapy are also verified by historical and observational evidence, with decreases in erythema, pruritus, and lichenification commonly seen over extended treatment courses [39]. Additionally, saltwater bath immersion before exposure to UVB has been shown to affect the minimal erythema dose, reflecting photoprotective or photomodulatory activity potentially a result of mineral interaction at the epidermal interface [40]. Individual variability in response according to the mineral composition of salt solutions, however, implies the need for mineral standardization in future photobalneotherapeutic regimens [41].

While the findings of this review are promising, certain limitations should be acknowledged to contextualize the evidence. The included studies varied in sample size, intervention protocols, and outcome measures, contributing to heterogeneity across results. Moreover, only a limited number of randomized controlled trials were available, with several studies being observational in nature.

The heterogeneity in intervention protocols, follow-up duration, and outcome measures precluded formal meta-analysis, limiting statistical synthesis but maintaining methodological transparency and consistency. Future randomized controlled trials with standardized methodologies and longer follow-up periods are warranted to confirm and expand upon the therapeutic potential of marine-based therapies in atopic dermatitis.

## Conclusions

Based on the evidence presented in this review, seawater treatments such as climatotherapy from the Dead Sea, balneotherapy from thermal springs, and mineral water treatments had significant clinical efficacy in reducing the severity of AD across different ages and severities of disease. Persistent improvement in validated scoring systems such as SCORAD, EASI, and o-SCORAD was seen in the different modalities, with some showing benefits in skin barrier function (e.g., reduced transepidermal water loss and increased stratum corneum hydration). In addition, other advantages such as reduced microbial colonization - specifically with regard to *Staphylococcus aureus *- and beneficial modulation of the skin microbiota were also reported with some of the treatments. Of note, there were low and transient side effects, thus supporting the overall safety profile of the treatments. The results varied with the type of water, frequency of treatment, as well as adjunctive interventions, but evidential and sustained responses were specifically noted with Dead Sea minerals and Comano spring water therapies. Seawater therapy would, therefore, seem to be a safe and promising non-pharmacologic adjunct or alternative to the overall treatment of atopic dermatitis, especially in patients seeking steroid-sparing alternatives. However, more standardized and inclusive trials are required to optimize dosing regimens and determine long-term benefits in heterogeneous population groups.
